# Supplementary material for: Effects of Artemisia macrocephala Jacquem on Memory Deficits and Brain Oxidative Stress in Streptozotocin-Induced Diabetic Mice
Source: Molecules. 2022 Apr 8;27(8):2399. doi: 10.3390/molecules27082399 (PMC9028531; doi:10.3390/molecules27082399)
Supplement: Supplementary file 1 [file molecules-27-02399-s001.zip › molecules-1619551-supplementary.pdf]

molecules

MDPI

Supplementary file

Effects of *Artemisia macrocephala* Jacquem on Memory Deficits and Brain Oxidative Stress in Streptozotocin Induced Diabetic Mice

Atiqul Bari [1], Syed Muhammad Mukarram Shah [1], Fakhria A. Al-Joufi [2], Syed Wadood Ali Shah [3,*], Mohammad Shoaib [3], Ismail Shah [4], Muhammad Zahoor [5,*], Muhammad Naeem Ahmed [6], Mehreen Ghias [3], Syed Muhammad Hassan Shah [7] and Atif Ali Khan Khalil [8]

Supplementary information

Table S1. Cholinesterase (AChE and BuChE) inhibitory activity of crude extract and fractions.

Sample Test	Conc. (µg/mL)	AChE		BChE	
		% Inhibition	IC_{50} (µg/mL)	% Inhibition	IC_{50} (µg/mL)
**Crd-Am**	6.25	25.09±0.91	**116.36±1.48**	20.60±0.93	**240.52±1.35**
	15.5	23.61±1.15		17.19±1.44	
	31.25	34.10±1.21		27.09±1.25	
	62.5	45.61±1.09		35.77±1.31	
	125	53.71±1.48		43.12±1.49	
	250	57.14±1.33		51.97±1.35	
	500	65.09±1.67		57.12±1.40	
	1000	69.24±1.41		61.43±1.33	
**nhex-Am**	6.25	09.96±0.76	**934.75±1.21**	13.77±0.83	**509.26±1.14**
	15.5	13.07±0.92		17.96±1.09	
	31.25	17.11±1.51		21.89±1.40	
	62.5	21.29±1.38		34.12±1.37	
	125	25.56±1.27		39.09±1.24	
	250	39.20±1.51		43.72±1.38	
	500	46.51±1.44		49.09±1.14	
	1000	53.49±1.21		54.12±1.61	
**Chl-Am**	6.25	29.88±1.03	**52.68±1.09**	26.12±0.99	**57.45±1.39**
	15.5	35.21±1.13		31.90±1.11	
	31.25	38.52±1.21		35.61±1.28	
	62.5	59.31±1.09		54.39±1.39	
	125	63.44±1.12		60.91±1.30	
	250	73.79±1.29		70.62±1.44	
	500	77.22±1.44		73.19±1.51	
	1000	86.91±1.38		80.14±1.34	
**Et-Am**	6.25	25.87±1.11	**75.19±1.02**	23.66±±1.03	**116.58±1.09**

	15.5	30.93±1.09		31.08±±0.85	
	31.25	35.10±0.96		35.86±±1.01	
	62.5	41.56±1.02		45.57±±1.14	
	125	61.41±1.17		53.61±±1.09	
	250	63.60±1.21		57.22±±1.36	
	500	70.23±1.19		67.08±±1.29	
	1000	78.12±1.33		74.21±±1.48	
	6.25	19.11±1.19		13.45±0.91	
	15.5	25.71±1.30		20.97±1.17	
	31.25	30.12±1.22		26.28±1.29	
**But-Am**	62.5	42.11±1.42	**254.66±1.34**	43.73±1.45	**265.33±1.41**
	125	44.43±1.50		43.98±1.35	
	250	49.09±1.34		47.11±1.41	
	500	57.12±1.41		56.30±1.33	
	1000	67.61±1.39		62.19±1.49	
	6.25	15.60±0.93		14.72±0.98	
	15.5	19.67±1.22		17.96±1.25	
	31.25	25.29±1.25		24.11±1.41	
**Aq-Am**	62.5	32.76±1.37	**502.71±1.28**	29.61±1.30	**513.66±1.33**
	125	34.81±1.20		32.97±1.21	
	250	42.87±1.39		41.96±1.37	
	500	49.73±1.28		48.67±1.33	
	1000	56.12±1.33		54.83±1.29	
	6.25	61.98±0.67		77.89±0.71	
	15.5	73.67±1.01		86.41±1.13	
	31.25	81.28±1.19		90.12±1.29	
**Donepezil**	62.5	85.12±1.31	**5.04±0.67**	95.80±1.47	**4.01±0.71**
	125	93.88±1.49		98.16±1.40	
	250	97.12±1.41		99.08±1.37	
	500	97.27±1.39		99.10±1.35	
	1000	98.10±1.53		99.77±1.30	

All values are expressed as Mean ± SEM.

Table S2. Effect of crude extract and fractions of *A. macrocephala* on mice in novel object recognition test (NORT)in short term memory.

Treatment/Dose		Sample Phase		Test Phase		DI (%)
		**Identical Object A1**	**Identical Object A2**	**Novel Object A1**	**Familiar Object A2**	
**Control**		**18.98±0.63**	18.31±0.73	18.71±0.45	16.98±0.53	52.42±1.88
**Diabetic**		8.97±0.70	7.79±0.86	6.39±0.52###	13.01±0.71	32.93±1.91
**Crd-Am**	**100mg**	14.71±0.78	14.97±0.80	14.31±0.68**	11.33±0.73	55.81±1.76
	**200mg**	15.11±0.46	15.03±0.78	14.39±0.79**	10.76±0.61	57.21±1.54
**nhex-Am**	**75mg**	14.89±0.72	15.31±0.56	10.76±0.68*	13.29±0.52	44.74±1.56
	**150mg**	14.83±0.83	14.87±0.70	10.91±0.91**	13.26±0.65	45.13±1.92
**Chl-Am**	**75mg**	14.91±0.84	15.01±0.77	14.54±0.81***	9.35±0.47	60.86±1.81
	**150mg**	14.73±0.91	15.12±0.52	15.05±0.48***	9.21±0.55	62.03±1.98
**Et-Am**	**75mg**	14.91±0.75	14.88±0.73	14.43±0.81**	11.33±0.73	57.76±1.71
	**150mg**	14.86±0.66	14.93±0.79	14.22±0.90**	10.23±0.79	58.16±1.96
**But-Am**	**75mg**	14.96±0.75	14.99±0.50	11.56±0.53*	13.11±0.73	46.85±1.77
	**150mg**	14.83±0.77	15.08±0.84	11.39±0.67*	13.27±0.59	46.18±1.69
**Aq-Am**	**75mg**	14.81±0.66	14.72±0.81	11.65±0.86**	12.49±0.60	48.26±1.73
	**150mg**	15.09±0.70	15.04±0.56	11.48±0.74*	12.91±0.74	47.07±1.78
**Donepezil**	**2mg**	14.98±0.90	14.64±0.61	14.92±0.84***	8.73±0.65	64.09±2.01
**Metformin**	**50mg**	14.69±0.83	15.08±0.56	15.07±0.79***	9.01±0.69	62.03±1.87

All values are expressed as Mean±SEM (n=6). Oneway ANOVA after which Dunnett's post hoc multiple comparison test. ###P<0.001 comparison of STZ treated diabetic (amnesic) group vs. normal control, *P<0.05, **P<0.01 and ***P<0.001 as comparison of STZ treated diabetic (amnesic) group vs. Donepezil, Metformin, crude extract and fractions-treated groups, using one way ANOVA followed by Dunnet comparison. Crude extract (Crd-Am), hexane (nhex-Am), chloroform (Chl-Am), ethyl acetate (Et-Am), butanol (But-Am) and aqueous (Aq-Am) fraction.

Table S3. Effect of crude extract and fractions of *A. macrocephala* on mice in novel object recognition test (NORT) in long term memory.

Treatment/Dose		Sample Phase		Test Phase		DI (%)
		Identical Object A1	Identical Object A2	Novel Object A1	Familiar Object A2	
**Control**		20.30±0.93	21.81±0.84	15.95±0.79	11.68±0.78	57.72±1.91
**Diabetic**		13.08±0.68	11.41±0.88	6.26±0.52$^{###}$	13.28±0.69	32.03±1.83
**Crd-Am**	**100mg**	18.43±0.87	16.73±0.79	14.85±0.87**	11.33±0.73	56.72±1.89
	**200mg**	17.23±0.88	19.06±0.72	17.01±0.77***	12.06±0.78	58.51±2.05
**nhex-Am**	**75mg**	18.31±0.97	16.64±0.89	10.74±0.68**	12.29±0.80	46.63±1.88
	**150mg**	17.06±0.62	19.40±0.62	10.99±0.89***	13.26±0.75	45.32±1.91
**Chl-Am**	**75mg**	18.50±0.70	16.65±0.74	17.42±0.77**	10.06±0.51	63.39±2.08
	**150mg**	17.56±0.67	19.02±0.58	19.21±0.89***	10.77±0.74	64.07±2.11
**Et-Am**	**75mg**	18.35±0.81	16.83±0.70	16.70±0.72**	11.35±0.81	59.53±1.97
	**150mg**	17.30±0.96	19.16±0.78	17.71±0.69***	11.33±0.85	60.98±1.89
**But-Am**	**75mg**	18.53±0.84	16.78±0.84	11.06±0.57**	12.11±0.63	47.73±1.82
	**150mg**	17.40±0.79	19.22±0.81	11.39±0.67***	12.31±0.62	48.06±1.88
**Aq-Am**	**75mg**	18.47±0.87	16.76±0.70	11.65±0.63**	12.16±0.67	48.93±1.85
	**150mg**	17.36±0.76	19.13±0.60	12.05±0.67***	12.11±0.66	49.87±1.79
**Donepezil**	**2mg**	16.45±0.83	17.95±0.63	23.94±0.84***	12.27±0.87	66.11±1.97
**Metformin**	**50mg**	18.23±0.88	17.13±0.68	17.12±0.87**	10.26±0.78	62.52±2.01

Mean ± SEM (n=6). Oneway ANOVA after which Dunnett's post hoc multiple comparison test. ###P<0.001 comparison of STZ treated diabetic (amnesic) group vs. normal control, *P<0.05, **P<0.01 and ***P<0.001 as comparison of STZ treated diabetic (amnesic) group vs. Donepezil, Metformin, crude extract and fractions-treated groups, using one way ANOVA followed by Dunnet comparison. Crude extract (Crd-Am), hexane (nhex-Am), chloroform (Chl-Am), ethyl acetate (Et-Am), butanol (But-Am) and aqueous (Aq-Am) fraction.

Table S4. Effect of Crd-Am and fractions on AChE and ACh level in brain.

Sample Test		AChE (µmoles of substrate hydrolysed / min / g tissue)	ACh (mmol/min/mg protein)
**Control**		12.01±1.13	16.21±1.33
**Diabetic**		28.92±1.23§§§	6.17±1.19‡‡‡
**Crd-Am**	**100mg**	16.11±1.08*	12.87±1.21*
	**200mg**	15.09±1.15**	13.33±1.26**
**Chl-Am**	**75mg**	14.01±1.01***	13.48±1.22**
	**150mg**	13.61±1.34***	13.92±1.45***
**Et-Am**	**75mg**	15.03±1.12**	13.09±1.39**
	**150mg**	14.98±1.27**	13.37±1.25**
**Donepezil**	**2mg**	12.38±1.36***	15.08±1.44***
**Metformin**	**50mg**	11.92±1.19***	15.17±1.51***

Mean±SEM (n = 6). Oneway ANOVA after which Dunnett's post hoc multiple comparison test. §§§ P<0 001, ‡‡‡P<0 001 comparison of STZ treated diabetic (amnesic) group vs. normal control, *P<0.05, **P<0.01 and ***P<0.001 as comparison of STZ treated diabetic (amnesic) group vs. Donepezil, Metformin, crude extract and fractions-treated groups, using one way ANOVA followed by Dunnet comparison. Crude extract (Crd-Am), chloroform (Chl-Am) and ethyl acetate (Et-Am) fraction.
